# The challenge of preventing gastric cancer in patients under surveillance for familial adenomatous polyposis

**DOI:** 10.1007/s10689-024-00438-4

**Published:** 2025-01-08

**Authors:** Hicham Bouchiba, Arthur S. Aelvoet, Nicole C. T. van Grieken, Lodewijk A. A. Brosens, Barbara A. J. Bastiaansen, Evelien Dekker

**Affiliations:** 1 Department of Gastroenterology and Hepatology, Amsterdam UMC, University of Amsterdam, Amsterdam Gastroenterology Endocrinology Metabolism, Cancer Center Amsterdam, Amsterdam, The Netherlands; 2 Department of Pathology, Amsterdam UMC, Vrije Universiteit Amsterdam, Cancer Center Amsterdam, Amsterdam, The Netherlands; 3 Department of Pathology, University Medical Center Utrecht, Utrecht University, Utrecht, The Netherlands

**Keywords:** Adenomatous polyposis coli, Stomach neoplasm, Endoscopy, Survival

## Abstract

Several extra-colonic manifestations, including duodenal polyposis and desmoid tumors, are well-described manifestations in familial adenomatous polyposis (FAP). More recently, an increase in gastric cancer diagnoses has been observed in FAP. This case series presents nine patients with FAP who were diagnosed with gastric cancer at our FAP expertise center, of whom eight were diagnosed between 2017 and 2023, while before 2017 the only diagnosis of gastric cancer was in 2001. Among the nine cases of gastric cancer, seven were located in the proximal stomach amidst carpeting fundic gland polyposis and two were located in the distal stomach. Despite ongoing advances in endoscopic technology, all patients were diagnosed during regular endoscopic surveillance, and six of the nine patients died within two years. We aim to raise awareness on gastric cancer risk in FAP patients and stress the urgent need of improved gastric surveillance strategies with timely detection of gastric cancer precursors.

## Introduction

Familial adenomatous polyposis (FAP) is characterized by colorectal polyposis resulting from a germline pathogenic variant in the *adenomatous polyposis coli* (*APC*) gene [1]. FAP patients undergo lifelong endoscopic surveillance and (procto)colectomy to prevent cancer development [2].

Until recently, FAP patients in Western countries were not considered to be at increased risk of developing gastric cancer[3, 4]. However, the incidence of gastric cancer in patients with FAP has increased over the past decade in a tertiary care center[5], although large epidemiological studies failed to identify an elevated risk of gastric cancer in this population[6, 7]. It is particularly concerning that these gastric cancers are diagnosed in patients who are under active endoscopic surveillance. They predominantly develop in areas of extensive (carpeting) fundic gland polyps (FGP) in the proximal stomach, which might hamper the identification of premalignant lesions or even cancers [4, 5].

This retrospective single-center case series presents the characteristics of all gastric cancer cases in our expert center. The aim is to increase awareness of the risk of gastric cancer and to guide optimization of surveillance strategies.

### Case series

Within our FAP cohort of 309 patients, nine (2.9%) developed gastric cancer. Table 1 summarizes the baseline characteristics of these patients, while Table 2 illustrates the endoscopic and histopathological characteristics.

**Table 1:** Baseline characteristics of FAP patients with gastric cancer

Patient	Year of gastric cancer diagnosis	Age at diagnosis	Sex	Germline *APC* pathogenic variant	Colectomy	History of duodenectomy	Highest Spigelman stage described	Duration of Upper-GI surveillance (months)	Number of Upper-GI endoscopies
1	2001	50	F	Unknown*	Proctocolectomy with ileostomy	No	IV	45	12
2	2017	65	F	Unknown*	Proctocolectomy with ileostomy	No	IV	–	–
3	2018	52	F	Exon 3	Proctocolectomy with IPAA	No	II	110	10
4	2018	47	F	Exon 6	Proctocolectomy with IPAA	No	II	2	2
5	2021	64	F	Exon 15 c.2805C > A; p.(Tyr935*)	Proctocolectomy with IPAA	Yes	IV	80	11
6	2021	53	M	Exon 15 ( c.2805 > A p.Tyr935X)	Proctocolectomy with ileostomy	Yes	II	117	13
7	2021	57	F	Exon 14 c.1866C > A p.Tyr622	Colectomy with IRA	No	III	176	19
8	2022	44	F	Exon 15 c2118_2126dup, p Ser713fs	Colectomy with IRA	No	II	198	7
9	2022	33	F	Exon 3	Colon in situ	No	II	165	7

*Unknown whether testing was performed

**Table 2:** Endoscopic and pathological characteristics

Patient	Gastric polyposis	Earlier diagnosis of gastric adenoma (highest degree of dysplasia)	Location	Gastric carcinoma visualized during EGD?	Time of last endoscopy until gastric carcinoma diagnosis (months)	Image of gastric carcinoma or gastric adenoma prior to diagnosis	Stage of gastric adenocarcinoma*	Survival after diagnosis
1	No image Mild FGPs	Adenomatous mucosa with severe dysplasia (2000)	Distal-antrum	Yes	1	None Pathology: infiltrating adenocarcinoma with severe dysplasia	T1N0M0 -Stage I	Alive – 23 years after diagnosis. Patient underwent total gastrectomy, duodenectomy and cholecystectomy
2	Carpeting FGPs	Low-grade dysplasia removed	Proximal-corpus	Yes	Unknown	Pathology: gastric adenocarcinoma	T3NxM1 -Stage IV	Deceased – year after diagnosis. Patient underwent total gastrectomy and duodenectomy
3	Carpeting FGPs	Tubular adenoma with low-grade dysplasia	Proximal-corpus	Yes	2	Pathology: gastric adenocarcinoma	TxNxM1—Stage IV	Deceased – one month after diagnosis
4	Carpeting FGPs	Pyloric gland adenoma with low-grade dysplasia	Proximal—cardia	No	10	Pathology: tubulovillous adenoma with low-grade dysplasia	T4N3M1 -Stage IV	Deceased – within one month after diagnosis
5	Carpeting FGPs	FGP with partial low-grade and high-grade dysplasia	Proximal—corpus	Yes	4	Pathology: FGP with high-grade dysplasia with adenocarcinoma and possible lymphangio-invasion	T1NxM1 -Stage IV	Deceased – 24 months after diagnosis. Year after gastrectomy patient developed pulmonary metastasis
6	mild FGPs	Tubular adenoma with high-grade dysplasia	Distal—near the anastomosis	Yes	16	Pathology: gastric adenocarcinoma	T1N0M0-Stage I	Alive – subsequent three endoscopies no dysplasia
7	Carpeting FGPs	Tubulovillous adenoma with low-grade dysplasia	Proximal -cardia/ distal esophagus	Yes	14	Pathology: gastric/esophagus adenocarcinoma	T1N0M0 -Stage I	Alive – subsequent two endoscopies no dysplasia
8	Carpeting FGPs	Tubular adenoma with low-grade dysplasia	Proximal—cardia	No	10	Pathology: Tubular adenoma with low-grade dysplasia	TxN1M1—Stage IV	Deceased – 2 months after diagnosis
9	Carpeting FGPs	Tubulovillous adenoma with high-grade dysplasia	Proximal—corpus	No	4	Pathology: Intramucosal adenocarcinoma	T1NxM1—Stage IV	Deceased – one year after diagnosis

*Based on the 8th edition of the TNM classification

**Patient 1** (Female, 50 y/o) is the only patient diagnosed with gastric cancer before 2017. She had mild FGPs. In 2001, she underwent surveillance esophagogastroduodenoscopy (EGD) with piecemeal endoscopic mucosal resection (pEMR) of an antral lesion, which revealed an adenocarcinoma within an area of high-grade dysplasia (HGD). Due to the gastric cancer diagnosis and severe duodenal polyposis (Spigelman IV), a combined total gastrectomy with prophylactic pancreas-preserving total duodenectomy was performed. At present, twenty-three years later, she is still cancer-free.

**Patient 2** (Female, 65 y/o), was referred to our center after being diagnosed with gastric adenocarcinoma in the body of the stomach within carpeting FGPs, visualized during surveillance. Due to extensive duodenal polyposis (Spigelman IV), a combined total gastrectomy and (prophylactic) duodenectomy was performed, revealing a poorly differentiated T3 gastric adenocarcinoma without lymph node involvement or distant metastases. Seven months after surgery, the patient presented with abdominal complaints and a CT-scan revealed a mass in the pelvis and a lesion in the liver. She underwent surgery of the pelvic mass and a biopsy of the liver lesion. Both revealed gastric adenocarcinoma metastasis. Palliative chemotherapy was initiated. She died one year after the gastric cancer diagnosis.

**Patient 3** (Female, 52 y/o), presented with acute hematemesis. EGD revealed an ulcerative lesion in the proximal stomach amidst carpeting FGPs. Biopsies confirmed gastric adenocarcinoma, and imaging showed liver metastases. Palliative systematic therapy was initiated. Surveillance had been performed four months prior to the gastric adenocarcinoma diagnosis, which did not reveal any gastric abnormalities other than carpeting FGPs. One month after diagnosis she passed away due to disease progression.

**Patient 4** (Female, 47 y/o) underwent surveillance EGD, which revealed a lesion suspicious for dysplasia in the body of the stomach. Endoscopic submucosal dissection (ESD) of the lesion was performed, revealing low-grade dysplasia (LGD). However, 8 months later she presented with abdominal complaints and weight loss. A CT-scan revealed metastases in the liver and lungs of unknown primary origin. Palliative care was provided and the patient died after two months. Autopsy revealed a T4 gastric adenocarcinoma located in the proximal stomach amidst carpeting FGPs, just below the Z-line contralateral to the scar of the previous ESD. The metastases had the same morphology as the gastric adenocarcinoma.

On surveillance EGD,** patient 5** (Female, 64 y/o) had a lesion suspicious for dysplasia in the gastric body within carpeting FGPs. Piecemeal EMR was performed, revealing high-grade dysplasia (HGD) and an area of poorly differentiated adenocarcinoma (T1) with suspicion of lymphangioinvasion. A CT-scan showed no lymphadenopathy or metastases. Following multidisciplinary team discussion, the patient underwent total gastrectomy. Histopathological examination did not reveal any residual carcinoma or HGD, and all resected lymph nodes were free of cancer. However, one year after surgery, a follow-up PET/CT-scan revealed multiple intrapulmonary metastases and lymphadenopathy. Palliative chemotherapy was initiated, and patient died one year later.

**Patient 6** (Male, 53 y/o) underwent a pylorus-resecting pancreatoduodenectomy 20 years before the diagnosis of gastric adenocarcinoma. Since this procedure, the patient experienced severe biliary reflux. In previous EGDs, 17 gastric adenomas were removed (LGD) from the remaining stomach. This patient did not have carpeting FGPs. During subsequent EGD, a dysplastic lesion near the gastro-jejunal anastomosis was detected and radically removed by ESD. Pathology revealed poorly differentiated, mucosal adenocarcinoma without lymphangioinvasion and imaging did not show metastasis. Patient is currently undergoing endoscopic and radiologic surveillance every six months. Almost three years after ESD, there are no signs of recurrence or metastases.

**Patient 7** (Female, 57 y/o) was diagnosed with gastric adenocarcinoma at the gastro-esophageal junction during surveillance. The patient had carpeting FGPs in the proximal stomach. Subsequently, endoscopic ultrasound was performed, which showed no involvement of lymph nodes. The lesion was removed by ESD, and histopathological examination confirmed an R0-resection of a well-to-moderately differentiated, mucosal adenocarcinoma without lymphangioinvasion. Currently, two years later, the patient undergoes surveillance every 6 months with no signs of recurrence.

**Patient 8** (Female, 44 y/o) presented with abdominal complaints. A CT-scan revealed metastases in both the liver and lungs. The patient received palliative therapy and passed away two months later. Autopsy revealed a well-to-moderately invasive gastric adenocarcinoma in the cardia with metastases and carpeting FGPs in the proximal stomach. A dysplastic lesion (LGD) was removed during previous surveillance.

**Patient 9** (Female, 33 y/o) is the daughter of patient 3 and had carpeting FGPs. During surveillance, two lesions were removed by pEMR. Histopathological examination revealed HGD and surveillance was intensified to three monthly. However, before the next surveillance, she presented with acute hip pain. An MRI revealed soft tissue invasion in the acetabulum. Biopsies confirmed adenocarcinoma, likely metastasis from the upper-gastrointestinal tract. PET-CT and subsequent EGD showed no evidence of gastric adenocarcinoma. The patient underwent a hemipelvectomy. Three months later, she presented with abdominal complaints and MRI revealed multiple liver metastases. Histopathologic revision of the gastric lesion with HGD demonstrated an intramucosal adenocarcinoma and molecular clonal relation with the liver metastasis. The patient received palliative systemic therapy and passed away one year after diagnosis due to disease progression.

## Discussion

In our cohort of 309 FAP patients, nine developed gastric cancer, of whom seven in the last decade and all under endoscopic surveillance. Six of these patients, all females with carpeting FGPs, died within two years from diagnosis due to metastasized disease. Among these patients, two were diagnosed with intramucosal adenocarcinoma, of which one without lymphangioinvasion. Despite this early stage, the disease had already metastasized.

The increase in gastric cancer diagnoses over time in FAP patients may be explained by two hypotheses. First, improved detection through advances in endoscopic techniques and greater (gastric) awareness during surveillance has likely led to more cases being identified. Earlier upper-gastrointestinal tract surveillance in FAP primarily focused on the duodenum, potentially resulting in missed or misclassified cases of gastric cancer. Second, increased life expectancy due to improved prevention of colorectal cancer in FAP may have exposed these individuals to a higher risk of developing other cancers, including gastric cancer. However, the median age at gastric cancer diagnosis in our cohort was 52 (range 33–65 year) suggesting that extended survival alone may not fully account for the observed risk.

Table 3 summarizes the reported series on gastric cancer in FAP [4, 5, 8–18]. Our findings are comparable to other series from Western countries [4, 5, 9–11, 13, 14]. Of the 37 gastric cancers with a reported location in Western-patients, 31 were located in the proximal stomach amidst carpeting FGPs. In contrast, FGPs were only present in 6/17 patients from Asian countries, if reported at all [15–18]. The location of the gastric adenocarcinomas was only reported in 6 patients and was proximal in 4 of those. These different characteristics can be attributed to a possible distinct etiology of gastric cancer, where in Japan and Korea higher incidences of gastric cancer in FAP are described, potentially related to more common *Helicobacter pylori* (*H. pylori)* infections in Asia [19, 20]. However, despite the overall higher incidence of gastric cancer in Asia, a Korean study reported a significantly elevated risk of gastric cancer in FAP patients compared to the general population. In our series, as well as three other Western series, *H. pylori* infection was not observed in any patients diagnosed with gastric cancer [10, 11, 21]. These findings suggest that alternative mechanisms may play a central role in the development of gastric cancer in FAP. Familial predisposition may also contribute to gastric cancer risk in FAP, as illustrated by two related patients (Patients 3 and 9) in our cohort.

**Table 3:** Review of gastric cancer cases in FAP

Authors/ years	Country	Patients with gastric cancer (n = 70)	Median age at diagnosis (range)	Sex, female %	Significant Spigelman stage III-IV, n (%)	Fundic gland polyposis, %	Location, proximal n %
Present series	Netherlands	9	52 (33–65)	88.9%	4 (44.4%)	77.8%	7 (77.8%)
Christenson et al./ 2024 [8]	USA	6	57 (48–64)	20%	Unknown	Unknown	Unknown
Varanese et al./ 2023 [9]	Italy	2	47/53	50%	Unknown	100%	2 (100%)
DelSignore et al./ 2023 [10]	USA	2	57/67	Unknown	Unknown	100%	2 (100%)
Wang et al./ 2022 [11]	USA	3	54 (29–66)	Unknown	Unknown	100%	3 (100%)
Cannon et al./ 2021 [12]	USA	7	55 (41–83)	43%	Unknown	Unknown	Unknown
Mankaney et al./ 2017 [5]	USA	10	58.5 (36–75)	60%	7 (70%)	100%	10 (100%)
Walton et al. / 2017 [4]	UK	8	52 (41–64)	62%	7 (88%)	100%	5 (62.5%)
Ravoire et al./ 2014 [13]	France	2	40/63	50%	Unknown	100%	1 (50%)
Garrean et al./ 2008 [14]	USA	1	61	0	Unknown	100%	1 (100%)
Tanabe et al./ 2020 [15]	Japan	1	Unknown	100%	Unknown	100%	1 (100%)
Shibata et al./ 2013 [16]	Japan	5	56 (49–72)	40%	Unknown	None had FGPs	3 (60)
Yamaguchi et al./ 2016 [17]	Japan	11	50.3 (+—15.0)	36.4%	Unknown	54.5% had no FGPs	Unknown
Park et al./ 1992 [18]	Korea	3	44 (35–55)	33.3%	Unknown	Unknown	Unknown

Timely detection of proximal gastric cancers, or ideally, its precursor lesions, in FAP patients seems to be a significant challenge. Recent efforts to enhance risk stratification include the SPACE (surveillance for pathology associated with cancer on endoscopy) criteria, which classify gastric polyps based on endoscopic features such as color, pit pattern, and surface morphology, aiding in the detection of high-risk lesions [22]. Shimamoto et al. further demonstrated that a wider distribution of FGPs significantly correlates with gastric neoplasm risk [23]. In our series, 5 out of 7 proximal gastric cancers were diagnosed during routine endoscopic surveillance, compared to 2 out of 10 patients in the series by Mankaney et al., highlighting the difficulties in detecting these cancers even under active surveillance in expert centers. [5]. Carpeting FGPs, present in all patients with proximal gastric cancer in both series, not only hamper the identification of (subtle) gastric dysplastic lesions and cancer but are also considered a risk factor for gastric cancer development [21, 23]. Additionally, large solitary FGPs (≥ 2 cm) and polypoid mounds have been identified as high-risk features associated with proximal gastric cancer [21]. Another alarming finding is that symptomatic patients and asymptomatic patients with cancer detected at surveillance share the same poor prognosis.

Historically, the intervals for EGD surveillance have been determined by the severity of duodenal disease using the Spigelman classification [24]. Gastric findings are not incorporated in this classification system. However, our findings demonstrate that gastric cancer also occur in patients with only mild duodenal polyposis and long surveillance intervals. In our series, four patients with gastric adenocarcinoma had a Spigelman stage II, and the series of Mankaney et al*.* showed similar findings [5]. Therefore, high-quality endoscopic examination of the stomach is important and gastric findings should be taken into account when determining the next surveillance interval. The European FAP Consortium has proposed a surveillance strategy that incorporates gastric findings and is currently being studied prospectively [25].

Little is known about the molecular etiology of gastric adenocarcinoma in FAP. One recent report of a proximal gastric adenocarcinoma in a FAP patient described a molecular pathway similar to the colonic adenoma-carcinoma sequence with somatic *KRAS* and *PIK3CA* mutations [15]. FGPs are the most common gastric polyps in FAP. While sporadic FGPs rarely show dysplasia [26], more than one-third of FAP-associated FGPs have been found to harbor LGD, with cases of HGD also reported [21, 27]. A study highlighted that FAP-associated FGPs frequently exhibit somatic *APC* alterations, including mutations at codons 1554–1556 and allelic loss at 5q, suggesting these changes may precede dysplasia [28]. Rare *APC* somatic changes, such as deletions at codons 1502–1517 and 1465, have been identified in FGPs with HGD [29]. In our series, seven out of nine patients with gastric cancer had a known germline *APC* variant, all located prior to codon 935. Further histopathological and molecular investigations of gastric carcinogenesis in FAP are necessary to identify potential precursor lesions and understand their progression to cancer. These findings can be used to optimize endoscopic surveillance with the ultimate goal of preventing gastric cancer in FAP.

This series provides detailed insights into gastric cancer development in FAP patients and raises awareness of this emerging risk. However, the single-center design and small sample size may limit the generalizability of the findings.

In conclusion, gastric cancer in FAP is poorly detected endoscopically and has a poor prognosis when diagnosed, despite relatively low T-stages. There is a clear need for a better understanding of its etiology and an improved endoscopic surveillance protocol that includes a thorough assessment of the stomach.

## Data Availability

No datasets were generated or analysed during the current study.
